# Nivolumab improved survival vs dacarbazine in patients with untreated advanced melanoma

**DOI:** 10.1186/1479-5876-13-S1-O6

**Published:** 2015-01-15

**Authors:** Georgina V Long, Victoria Atkinson, Paolo A Ascierto, Benjamin Brady, Caroline Dutriaux, Michele Maio, Laurent Mortier, Jessica C Hassel, Piotr Rutkowski, Catriona McNeil, Ewa Kalinka-Warzocha, Kerry J Savage, Micaela Hernberg, Celeste Lebbé, Julie Charles, Catalin Mihalcioiu, Vanna Chiarion-Sileni, Cornelia Mauch, Henrik Schmidt, Dirk Schadendorf, Helen Gogas, Christine Horak, Brian Sharkey, Ian M Waxman, Caroline Robert

**Affiliations:** 1 Melanoma Institute Australia, University of Sydney, and Mater Hospital, Sydney, Australia; 2 Princess Alexandra Hospital, Wooloongabba & Gallipoli Medical Research Foundation, Greenslopes Private Hospital Queensland, Australia; 3 Istituto Nazionale Tumori Fondazione Pascale, Naples, Italy; 4 Cabrini Health, Melbourne, Australia; 5 Hôpital Saint André CHU, Bordeaux, France; 6 University Hospital of Siena, Siena, Italy; 7 Hôpital Claude Huriez, Lille, France; 8 University Hospital Heidelberg & National Center for Tumor Diseases, Heidelberg, Germany; 9 Maria Sklodowska-Curie Memorial Cancer Center & Institute of Oncology, Warsaw, Poland; 10 Chris O'Brien Lifehouse, The Melanoma Institute Australia & Royal Prince Alfred Hospital, Camperdown, Australia; 11 Wojewodzki Szpital Specjalistyczny im. M. Kopernika, Lodz, Poland; 12 British Columbia Cancer Agency, Vancouver, Canada, Canada; 13 Department of Oncology, Helsinki University Central Hospital, Helsinki, Finland; 14 APHP Dermatology and CIC Hôpital Saint Louis Paris 7 University, INSERM 976, Paris, France; 15 Grenoble University Hospital Grenoble France - INSERM U823, Joseph Fourier University, Grenoble, France; 16 Royal Victoria Hospital, Alberta, Canada; 17 Oncology Institute of Veneto IRCCS, Padua, Italy; 18 Department of Dermatology, University Hospital Cologne and CIO Köln Bonn, Germany; 19 Department of Oncology, Aarhus University Hospital, Aarhus, Denmark; 20 Department of Dermatology, University of Essen, Essen, Germany; 21 University of Athens Medical School, Laiko General Hospital, Athens, Greece; 22 Bristol-Myers Squibb, Lawrenceville, NJ, USA; 23 Bristol-Myers Squibb, Wallingford, CT, USA; 24 Gustave, Roussy and INSERM U981, Villejuif-Paris-Sud, France

## Background

The phase 1 study of nivolumab, a fully human IgG4 programmed death-1 (PD-1) immune checkpoint inhibitor monoclonal antibody, showed promising antitumor activity in patients with advanced melanoma.

## Materials and methods

This phase 3 study compared nivolumab vs dacarbazine in treatment-naïve patients with BRAF wild-type metastatic melanoma. Patients were randomized 1:1 to receive nivolumab 3 mg/kg every 2 weeks (Q2W) + placebo Q3W (n = 210) or dacarbazine 1000 mg/m2 Q3W + placebo Q2W (n = 208) until disease progression or unacceptable toxicity. Randomization was stratified by M-stage and programmed death ligand-1 (PD-L1) status. The primary endpoint was overall survival (OS). Patients were followed for up to 16.7 months at the time of data cutoff, which occurred 5.2 months after the first visit of the last patient randomized.

## Results

The hazard ratio (HR) for death was 0.42 (99.79% CI 0.25–0.73; *P* < 0.0001) in favor of nivolumab, with 1-year OS rate 73% (95% CI, 66%–79%) for nivolumab vs 42% (95% CI, 33%–51%) for dacarbazine. Median OS was not reached for nivolumab and was 10.8 months for dacarbazine. Median progression-free survival (PFS) was 5.1 months for nivolumab and 2.2 months for dacarbazine (HR for death or progression 0.43, 95% CI 0.34–0.56; *P* < 0.0001). Objective response rate was 40% (84/210) vs 14% (29/208) for nivolumab and dacarbazine, respectively (*P* < 0.0001). Median duration of response was not reached for nivolumab and 6 months for dacarbazine. At the time of data cutoff, responses were ongoing in 86% (72/84) of nivolumab and 52% (15/29) of dacarbazine responders. PD-L1 positivity (using a 5% tumor cell surface staining cutoff) appeared to be associated with improved OS in the nivolumab arm (85% of PD-L1+ and 71% of PD-L1-/indeterminate patients alive at the time of last follow-up). Both PD-L1+ and PD-L1-/indeterminate patients receiving nivolumab had improved OS vs dacarbazine (un-stratified HR 0.30, 95% CI, 0.15–0.60 in PD-L1+ patients; 0.48, 95% CI, 0.32–0.71 in PD-L1-/indeterminate patient, both in favor of the nivolumab arm). The most common nivolumab-related adverse events (AEs) were fatigue, pruritus, and nausea. Drug-related grade 3–4 AEs were reported in 12% vs 18% of patients receiving nivolumab vs dacarbazine, respectively. AEs led to discontinuation in 7% and 12% of dacarbazine- vs nivolumab-treatment patients, respectively.

## Conclusions

Compared to dacarbazine, nivolumab significantly improved OS and PFS in previously untreated patients with BRAF wild-type metastatic melanoma with an acceptable safety profile.

## Clinical trial registration number

NCT01721772

